# Corporate political activity in the context of unhealthy food advertising restrictions across Transport for London: A qualitative case study

**DOI:** 10.1371/journal.pmed.1003695

**Published:** 2021-09-02

**Authors:** Kathrin Lauber, Daniel Hunt, Anna B. Gilmore, Harry Rutter

**Affiliations:** 1 Department for Health, Tobacco Control Research Group, University of Bath, Bath, United Kingdom; 2 SPECTRUM Consortium (Shaping Public Health Policies to Reduce Inequalities and Harm), Edinburgh University, Edinburgh, United Kingdom; 3 Independent Researcher and Freelance Health Policy Consultant, Bath, United Kingdom; 4 Department of Social and Policy Sciences, University of Bath, Bath, United Kingdom; Carolina Population Center, UNITED STATES

## Abstract

**Background:**

Diets with high proportions of foods high in fat, sugar, and/or salt (HFSS) contribute to malnutrition and rising rates of childhood obesity, with effects throughout the life course. Given compelling evidence on the detrimental impact HFSS advertising has on children’s diets, the World Health Organization unequivocally supports the adoption of restrictions on HFSS marketing and advertising. In February 2019, the Greater London Authority introduced novel restrictions on HFSS advertising across Transport for London (TfL), one of the most valuable out-of-home advertising estates. In this study, we examined whether and how commercial actors attempted to influence the development of these advertising restrictions.

**Methods and findings:**

Using requests under the Freedom of Information Act, we obtained industry responses to the London Food Strategy consultation, correspondence between officials and key industry actors, and information on meetings. We used an existing model of corporate political activity, the Policy Dystopia Model, to systematically analyse arguments and activities used to counter the policy. The majority of food and advertising industry consultation respondents opposed the proposed advertising restrictions, many promoting voluntary approaches instead. Industry actors who supported the policy were predominantly smaller businesses. To oppose the policy, industry respondents deployed a range of strategies. They exaggerated potential costs and underplayed potential benefits of the policy, for instance, warning of negative economic consequences and questioning the evidence underlying the proposal. Despite challenging the evidence for the policy, they offered little evidence in support of their own claims. Commercial actors had significant access to the policy process and officials through the consultation and numerous meetings, yet attempted to increase access, for example, in applying to join the London Child Obesity Taskforce and inviting its members to events. They also employed coalition management, engaging directly and through business associations to amplify their arguments. Some advertising industry actors also raised the potential of legal challenges. The key limitation of this study is that our data focused on industry–policymaker interactions; thus, our findings are unable to present a comprehensive picture of political activity.

**Conclusions:**

In this study, we identified substantial opposition from food and advertising industry actors to the TfL advertising restrictions. We mapped arguments and activities used to oppose the policy, which might help other public authorities anticipate industry efforts to prevent similar restrictions in HFSS advertising. Given the potential consequences of commercial influence in these kinds of policy spaces, public bodies should consider how they engage with industry actors.

## Abstract 2

In a qualitative case study, Kathrin Lauber and colleagues examine the nature of corporate political activity in the context of unhealthy food advertisement restrictions across transport for London.

## Author summary

**Why was this study done?:**

- One in 3 children in England have obesity or overweight. These children are likely to be at higher risk for a range of health problem as adults.
- In light of unequivocal evidence that marketing and advertising influence children’s diets, in 2019, the Greater London Authority introduced a ban on advertising of foods and nonalcoholic beverages high in fat, sugar, and/or salt (HFSS) across the Transport for London (TfL) estate, one of the most valuable out-of-home advertising estates. This was one of the first policies of this kind.
- In other policy settings, restrictions on advertising have faced strong opposition from commercial actors, yet, to our knowledge, this has not been examined comprehensively in the United Kingdom.

**What did the researchers do and find?:**

- To explore if and how food and advertising industry actors opposed the advertising restrictions in the lead-up to their implementation, we analysed their submissions to the London Food Strategy consultation and their correspondence with officials, obtained via Freedom of Information requests.
- Employing a conceptual model based on research covering decades of tobacco industry activity, we mapped key arguments and activities used to oppose the ban.
- The majority of food and advertising industry respondents to the London Food Strategy consultation, in particular multinational companies and business associations, opposed the policy. Most food and advertising actors who supported the policy were smaller businesses.
- Opposition was justified by underplaying the potential benefits of the policy and by exaggerating its potential negative impacts and costs. Though opposing industry actors challenged the substantial evidence underpinning the policy, they used evidence only sparingly to justify their claims.
- A number of food and advertising companies engaged with the policy process through numerous meetings and calls, both directly and via business groups, which amplified opposing voices. Despite being involved in the stakeholder consultation, commercial actors attempted to increase their access to the policy process and officials, for example, by applying to join the London Child Obesity Taskforce. Moreover, 3 advertising companies hinted at the potential for a legal challenge.

**What do these findings mean?:**

- Although our findings are unable to provide a comprehensive picture of attempts to counter the policy, other public authorities looking to introduce unhealthy food advertising restrictions in public estates can anticipate similar strategies to those we observed. Our findings may help them evaluate if and how they want to engage with commercial actors and, where they do so, to make a more objective assessment of consultation responses.

## Background

Unhealthy diets are a major cause of mortality and morbidity worldwide [1]. In England, over one-third of children in year 6 (age 10 to 11) have obesity or overweight, with a more than 2-fold increase in prevalence in the most deprived compared to the most affluent areas [2]. Children and adolescents with obesity are likely to become adults with obesity [3], at risk of a range of noncommunicable diseases [4,5]. Ultraprocessed foods and soft drinks have been directly linked to obesity and noncommunicable diseases such cancer and cardiovascular disease [6–12] and constitute over 65% of the calories consumed by children in the United Kingdom (UK) [13]. Despite increasing concern and action over recent decades, no country has been successful at reducing its obesity prevalence [14].

### Obesity and advertising

According to the World Health Organization, the evidence that exposure to marketing of foods high in fat, sugar, and/or salt (HFSS) influences children’s diets is “unequivocal” [15]. Unhealthy food marketing and advertising contribute to obesity in children and adolescents by shaping dietary attitudes and behaviours, including so-called “pester power” to persuade parents to purchase certain foods, and increasing consumption of such foods [16–22], particularly advertised brands [22]. Low-income and minority communities are disproportionately exposed to out-of-home food advertising [23–26], which is associated with higher levels of obesity [25].

To protect children from harmful advertising, pressure is mounting on countries across the world to regulate commercial practices [27]. Globally, restricting the advertising of unhealthy commodities on government-owned infrastructure, including public transport, is an emerging policy lever [28–30]. The UK government recently announced a set of new measures to achieve the ambitious aim of halving childhood obesity by 2030. For HFSS advertising, these include a 21:00 television watershed and a total online ban [31]. Hitherto, there has been little statutory regulation in this area. HFSS adverts during and around broadcast programmes of “particular appeal” to children under 16 were banned in 2007 by Ofcom, an independent statutory body [32]. This is governed in co-regulation [33] with the advertising industry and enforced by the Advertising Standards Authority (ASA) and the Committees of Advertising Practice, bodies funded by the advertising industry that also self-regulate non-broadcast advertising (see Box 1).

**Box 1. Advertising self- and co-regulation in the UK**

**The ASA, CAP, and BCAP**

The Committees of Advertising Practice are responsible for writing and maintaining codes of practice—including, but not limited to, HFSS advertising [40]. Television and radio advertising licensed by Ofcom is covered by the UK Code of Broadcast Advertising (BCAP Code) [41], while the UK Code of Non-broadcast Advertising and Direct and Promotional Marketing (CAP Code) [42] covers non-broadcast advertising, sales promotions, and direct marketing. After a 2016 consultation, the CAP Code, which also applies to outdoor adverts across TfL, was updated to prohibit HFSS advertising in children’s media and all media where children under 16 make up over 25% of the audience [42]. The ASA is responsible for the enforcement and compliance monitoring of the CAP and BCAP Codes.

**Food industry self-regulation**

Food and beverage companies have also produced their own marketing codes, ranging from individual company pledges, such as those made by PepsiCo [43], to broader voluntary agreements between companies, for instance, the International Food and Beverage Alliance’s *Global Policy on Marketing Communications to Children* [44] and the *EU Pledge*, a self-regulatory initiative by food and beverage companies and the World Federation of Advertisers [45–47]. Overall, independent assessments of voluntary initiatives suggest that they are unlikely to be effective enough in reducing children’s exposure to unhealthy marketing [15,46,48–54], whereas industry evaluations tend to report high compliance levels and positive impacts on children’s environments [15,34].

While co-regulation appears more effective than self-regulation [34], findings on the effect of the UK co-regulatory regime are mixed. A 2010 Ofcom evaluation of the broadcast HFSS restrictions concluded that HFSS adverts had been “eliminated during children’s airtime,” yet the overall volume of HFSS advertising throughout the day increased and children saw only 1% less HFSS advertising during adult airtime [32]. Academic research found that the proportion of HFSS adverts seen by children increased from 43% before the rules came into force to 56% 6 months after their introduction [35]. The authors attributed this to a shift in spending towards other advertising channels, highlighting the need for comprehensive measures. In light of a growing evidence base supporting marketing restrictions [36,37], the World Health Organization and UNICEF recommend that a regulatory system to protect children from advertising should be comprehensive, covering the full range of advertising mediums and techniques children are exposed to, and use the UN definition of children up to 18 years [15,38,39].

### The Transport for London HFSS advertising restrictions

London is home to nearly 9 million people, over 2 million of whom are under 18 [55]. In the last 5 years, none of London’s 33 Boroughs saw a reduction in overweight or obesity rates among year 6 pupils [56]. As part of its mission to halve childhood obesity by 2030, the Greater London Authority (GLA) announced on 23 November 2018 that it would prohibit advertising of HFSS products across Transport for London (TfL), one of the most valuable out-of-home advertising estates that covers 1.5 billion passenger journeys a year [57,58]. While residents aged 25 to 44 and 45 to 59 make up the highest number of daily trips, a 2012 report suggested that nearly 80% of Londoners aged 11 to 15 use the bus at least once a week [59].

The policy, which came into force on 25 February 2019 [60], is among the first of its kind globally and goes further than the CAP code, which posits that outdoor adverts should not be targeted at an audience that consists to more than 25% of under-16s [61], and a similar measure in Amsterdam [62] where unhealthy food advertisements targeting children have been prohibited on the metro and other council-owned sites since January 2018. Several UK local authorities, most recently Bristol [63], have already followed suit, and such advertising restrictions are likely to be replicated more widely over the next years.

The advertising restrictions were consulted on from 11 May to 5 July 2018 as part of the London Food Strategy [64]. Alongside the draft Strategy, the GLA published a 2-page background paper on the proposed advertising restrictions [65]. The measure was widely supported by the public health community [66], and 82% of citizens who responded to the public consultation welcomed the restrictions, while the majority of the opposition came from food, beverage, and advertising industries [64]. The final policy prohibits direct and incidental advertising of HFSS foods and nonalcoholic beverages, as defined by the Department of Health Nutrient Profiling Model [67], “on all modes of transport controlled by TfL, including the Underground, Overground, London buses, TfL Rail, trams and river services” [66]. Moreover, it only permits food and beverage, restaurant, takeaway, and delivery companies to place adverts, “which promote their healthier [non-HFSS] products, rather than simply publicising brands” [66].

Policy development and implementation were led by a task-and-finish group within the GLA. The London Food Board, originally established in 2004, was tasked with advising the Mayor and the GLA on the London Food Strategy and played an important part in initiating the inclusion of advertising restrictions [68]. In March 2018, the London Child Obesity Taskforce (“Taskforce” hereafter) was established to advise the Mayor. Though its aims include work on advertising [69], the Taskforce was not involved in the development or implementation of the advertising restrictions. No multinational food or advertising corporations were directly represented on the Taskforce and the London Food Board as of April 2021. The Taskforce’s Chair stepped down from his role as founder of a baby food company in 2018 [70], and the Food Board includes a representative from the British Retail Consortium [68].

### Commercial opposition to public health regulation

One key barrier to effective action on healthier diets is industry interference [71,72]. Attempts by corporations to delay, weaken, and avert marketing regulation have been documented systematically in the cases of tobacco [73] and alcohol [74]. Although there is a growing body of literature mapping corporate political activity of major ultraprocessed food industry actors within select countries [75–84], efforts by commercial actors to undermine food and nonalcoholic beverage marketing regulation have not been scrutinised comprehensively. We aim to address this gap by investigating such a policy case, to our knowledge for the first time, in the UK and at the subnational level. Specifically, this study sought to answer the following question: How did food and advertising industry actors oppose the TfL HFSS advertising restrictions while the policy was being developed?

In doing so, we take a critical approach that conceptualises industry political practices as a mechanism through which corporations shape health outcomes or, in other words, a “commercial determinant of health” [85]. Thus, we concentrate on the practices of opponents of public health regulation, rather than those of public health advocates. Civil society and other health actors were involved throughout the development of the advertising restrictions [86], but they are not the focus of this study.

## Methods

To study food (including nonalcoholic beverage) and advertising industry conduct in the context of the TfL advertising restrictions, we employed a qualitative, heuristic case study approach [87], analysing multiple types of data using an existing model of corporate political activity, the Policy Dystopia Model (PDM) [88]. The University of Bath Research Ethics Approval Committee for Health granted ethical approval for this research (EP 18/19 068).

### Data collection

Where internal industry documents are not widely available, Freedom of Information requests have emerged as a method to collect data, which enable valuable insights into corporate interactions with public institutions [89–95]. We submitted iterative rounds of requests to the GLA, which led the policy development, as well as TfL and Public Health England, which were involved in the process (see S1 Table for details). Our requests covered consultation responses, correspondence, and information on meetings. Firstly, we asked for all food and advertising industry responses to the London Food Strategy consultation, none of which were initially published by the GLA. Secondly, we requested correspondence and information on meetings between officials and industry. As Freedom of Information exemptions and cost limits restrict the type and amount of data that can be accessed [96], we focused requests for correspondence and meeting information on the time span of April 2018 to January 2019—from before the consultation launch until just before the policy came into force—and a number of key food and advertising industry actors. We identified the latter through publicly available materials such as the consultation summary report [64] and informal discussion with experts.

### Analysis

Each consultation response we received was initially assessed for the following: (a) the nature of the submitting organisation (type of organisation, sector: for detailed classifications, see Table 2) and (b) whether it supported or opposed the advertising restrictions, or did not issue a (clear) opinion. Only responses by food and advertising industry respondents that opposed the advertising restrictions were included in our further analysis of discursive and instrumental strategies. Sections where respondents discussed other topics included in the London Food Strategy consultation, such as food waste and skills, were not included in the analysis.

Our analysis was rooted in a hermeneutic approach that carefully considered the meaning and context in which documents are produced [97,98]. Relevant consultation responses and all obtained correspondence leading up to the policy introduction in February 2019 were read and reread before we started coding.

**Fig 1:**
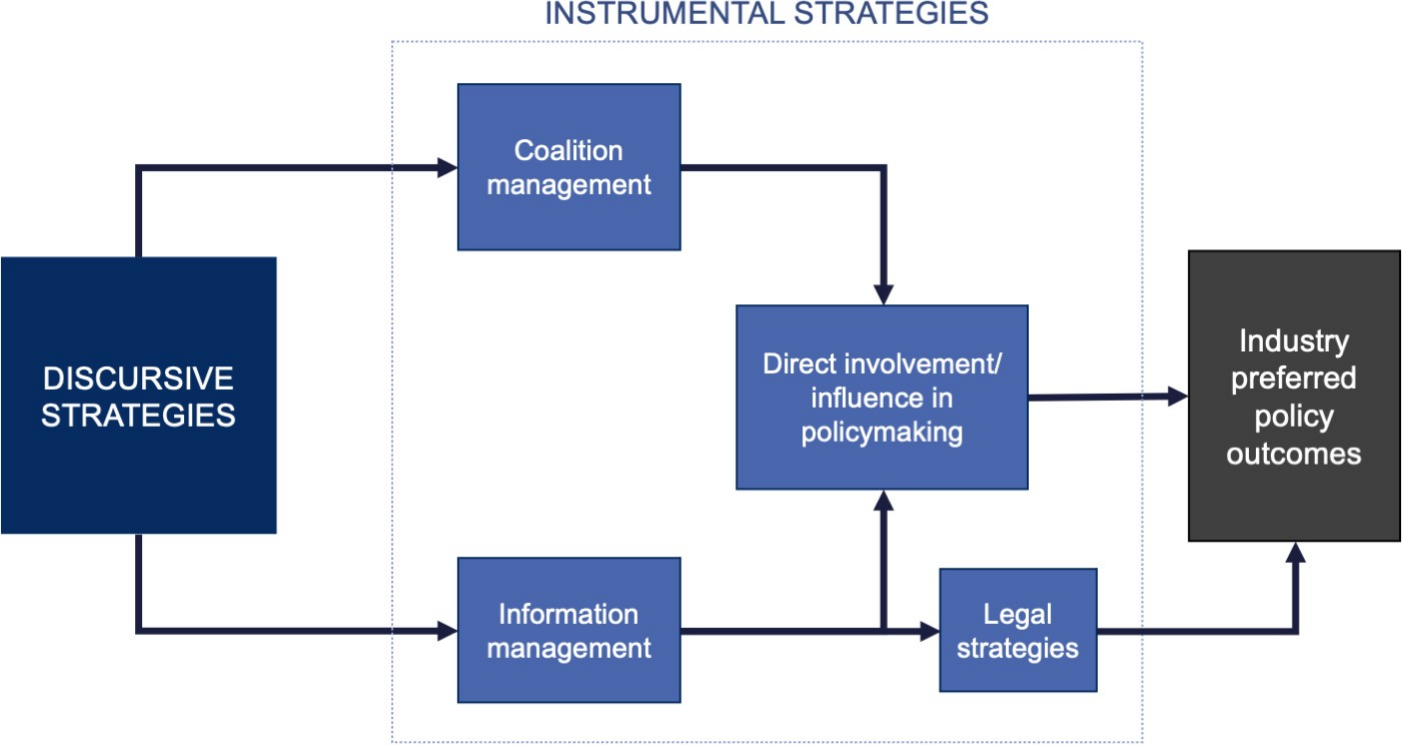
The Policy Dystopia Model [88].

We used the PDM [88]—a tool based on 2 systematic reviews of tobacco industry political activity [73,99]—as a lens for our analysis. The PDM posits that corporations construct a narrative that proposed policies will fail and lead to undesirable consequences (“dystopia”) and use a range of activities to distribute this narrative and achieve their preferred policy outcomes. As outlined in Fig 1 and Table 1, the hierarchical model is structured into discursive (argument-based) and instrumental (action-based) strategies. These strategies are further subdivided into “arguments” and “techniques,” respectively. Previous research using the PDM to map food [77,100] and alcohol [101] industry political activity has established that it is highly relevant to and can be used to study industries other than tobacco. We adopted the structure and core strategies of the PDM to guide our analysis. Moreover, we categorised overall positions against the advertising restrictions using industry policy aims from the PDM: delay, weaken, defeat. This may, for example, substantiate as requests to extend the implementation period, exempt products otherwise included, or not regulate advertising at all, respectively.

**Table 1:** Instrumental and discursive strategies from the PDM [88].

**Discursive strategies**	
**Expand or create potential costs**	
*Unanticipated costs to economy and society*	Exaggerating potential negative consequences of the proposed policy on the economy and (parts of) society.
*Unintended costs to public health*	Warning that the policy may have unintended negative consequences on public health.
*Unintended benefits to undeserving groups*	Arguing that the policy may result in unintended benefits to undeserving individuals or groups.
**Contain or deny potential benefits**	
*Intended public health benefits*	Claiming that the proposed policy is unlikely to have the intended public health benefits.
*Expected costs to industry*	Downplaying potential costs to own industry (while emphasising cost to other, more deserving groups such as small businesses).
**Instrumental strategies** ^**1**^	
**Coalition management**	Building or managing alliances with other companies or societal actors to establish alternative platforms for arguments. Industry participation in such coalitions primarily involves monetary contributions and may vary in transparency.
**Information management**	Producing and disseminating industry-favourable information while suppressing and undermining information in support of the policy. Information includes, but is not limited to, scientific evidence.
**Direct involvement and influence**	Access to, and representation or involvement in the policy process, including direct lobbying of policymakers.
**Legal strategies** ^**2**^	Legal action or the threat thereof.

^1^The PDM includes a subsidiary instrumental strategy—illicit trade—which was not included in our analysis as illicit trade has not been a prevalent topic in food policy debates.

^2^This strategy is called “litigation” in the original PDM. We use the term “legal strategies” to more clearly include not only legal action but also threats thereof.

PDM, Policy Dystopia Model.

#### Discursive strategies

We identified discursive strategies based on the included consultation responses. First, 2 members of the research team (KL and DH) conducted an initial round of open, thematic coding of 14 sample responses. Based on this, a list of key arguments was developed in discussion between the coders, grouped under the discursive strategies of the PDM, and applied to the complete set of included consultation responses.

#### Instrumental strategies

We analysed all included consultation responses and all correspondence between commercial actors and officials to identify instrumental strategies. Coding initially focused on identifying actions as the smallest unit of analysis, for instance, “sharing evidence with officials” or “requesting a meeting.” These actions were then collated into higher-order techniques, which, in turn, were grouped under the PDM instrumental strategies. Additionally, we extracted each instance where evidence—defined in a broad sense to include formal as well as informal sources such as reports—was referenced to support arguments around the advertising restrictions.

KL coded all data and DH second-coded one-third of the data to identify discursive and instrumental strategies. Interpretive discrepancies were resolved in discussion between the coders, and the analysis of instrumental and discursive strategies refined accordingly. Discussions within the wider research team helped to further refine the overall analysis. Analyses were conducted in NVivo 12 [102].

## Results

In response to our Freedom of Information requests, we obtained 38 industry consultation responses, 216 pages of emails between industry and officials from the GLA and TfL, and information on meetings (S1 Table). The average time from request to a final response was 37 working days. Sections of emails and submissions were redacted, predominantly invoking the Commercial Interests exemption to the Freedom of Information Act [96]. The GLA consulted with some respondents before releasing submissions or correspondence relating to them and withheld some information as a result. In the sections below, we provide an overview of the consultation responses before we move on to present the main results of our analysis, structured into discursive and instrumental strategies.

### London Food Strategy consultation responses

Eleven of the food and advertising industry responses obtained either supported the restrictions or did not express a clear opinion and were thus excluded from the analysis of discursive and instrumental strategies. Supporters were predominantly smaller companies which, in summary, adopted the position that restricting HFSS advertising would be an important step towards facilitating healthier diets (see S2 Table for details). The remaining 27 consultation responses, which opposed the policy (13 food companies/associations, predominantly involved in the manufacture/sale of ultraprocessed foods; 13 advertising companies/associations; one representing advertisers, including food companies), formed the basis of our analysis, alongside the correspondence and meeting information we obtained. Seven of 9 opposing food companies and 5 of 9 opposing advertising companies were members of at least one business association that responded to the consultation. Notably, almost all commercial actors suggested either voluntary alternatives or modifications to the policy in line with their own interests: Juice-producing companies, for instance, called for juice-based drinks to be exempt from the restrictions (Table 2). Proposed alternatives included adding healthy messaging to adverts, restricting HFSS advertising around schools, or limiting HFSS advertising on digital screens during times when more children travel.

**Table 2:** List of organisations that opposed the advertising restrictions as proposed in the draft London Food Strategy consultation. Policy aims regarding the proposed advertising restrictions are categorised based on the PDM—defeat (no advertising restrictions), delay (delayed implementation), weaken (advertising restrictions in a weaker form than proposed).

Submitting organisation	Type^1^	Sector^2^	BA membership^3^	Stance on advertising restrictions	Summary of proposed alternatives/modifications
FDF	BA	Food/beverage general	-	Defeat	No alternatives proposed.
BSDA	BA	Food/beverage general	-	Weaken	Suggests that lower-sugar drinks and fruit/vegetable-based drinks should be exempt from advertising restrictions (in line with the Soft Drinks Industry Levy).
BTC	BA	Foodservice	-	Unclear	Supports Just Eat’s position.
Dairy UK	BA	Food/beverage general	-	Weaken	Suggests that products containing over 75% milk, cheese or yogurt should be exempt from advertising restrictions.
ISBA	BA	Cross-sectoral (advertisers, including food companies)	Advertising Association	Defeat	Suggests that technology should be used to minimise HFSS advert exposure at times when children travel.
Innocent	Company	Food/beverage production	FDF, BSDA	Weaken	Suggests that fruit juice and smoothies should be exempt from advertising restrictions.
Just Eat	Company	Food delivery	BTC, ISBA	Defeat	Proposes voluntary alternatives, for instance, using technology to minimise HFSS advert exposure at times when children travel (jointly with Deliveroo).
Dominos	Company	Foodservice	ISBA	Defeat	Proposes voluntary alternatives, for instance, adding health messaging to HFSS adverts.
KFC	Company	Foodservice	ISBA	Defeat	Proposes voluntary alternatives, for instance, a “Schools Pact” partnership to promote “heathier choices” for children.
Lucozade Ribena Suntory	Company	Food/beverage production	FDF	Defeat	Proposes a focus on working with brands to promote healthy behaviour instead of banning unhealthy adverts, alongside enhanced self-regulation.
McDonald’s	Company	Foodservice	ISBA	Defeat	Proposes a partnership approach to reducing HFSS advertising and a focus on “nudges” and information campaigns.
PepsiCo UK	Company	Food/beverage production	FDF, BSDA, ISBA	Weaken	Suggests that fruit juice and smoothies should be exempt from advertising restrictions.
Subway	Company	Foodservice	-	Defeat	No alternatives proposed.
Uber Eats	Company	Food delivery	-	Defeat	Proposes voluntary alternatives, for instance, using technology to minimise HFSS advert exposure at times when children travel.
Outsmart	BA	Advertising	Advertising Association	Defeat	Proposes voluntary alternatives, for instance, health promotion campaigns.
Advertising Association	BA	Advertising (and cross-sectoral advertisers)	-	Defeat	No alternatives proposed.
IPA	BA	Advertising	Advertising Association	Defeat	Urges the Mayor to jointly explore alternative approaches with advertising agencies.
ASA System	Self-regulatory body	Advertising	-	Defeat	No alternatives proposed.
Clear Channel UK Ltd	Company	Advertising	Outsmart	Defeat	Proposes voluntary alternatives, for instance, health promotion campaigns and using technology to minimise HFSS advertising exposure at times when children travel.
Exterion Media UK Limited	Company	Advertising	-	Defeat	Endorses Outsmart response. Proposes health promotion campaigns across the TfL estate.
JC Decaux	Company	Advertising	Outsmart	Defeat	Proposes to increase healthy messaging and a targeted exclusion zone for HFSS advertising in a 100-m radius around schools.
Kinetic Worldwide	Company	Advertising	IPA	Defeat	Suggests expanding self-regulatory mechanisms and harnessing advertising space for healthy messaging.
Outdoor Plus	Company	Advertising	-	Defeat	Endorses Outsmart response. Proposes health promotion campaigns and limiting adverts targeted at children.
Primesight Limited	Company	Advertising	-	Defeat	Proposes voluntary alternatives, for instance, traffic light/Treatwise labelling on HFSS adverts.
Talon Outdoor	Company	Advertising	IPA	Defeat	Proposes voluntary alternatives, for instance, investment into healthy messaging and “Food Aware” notices on adverts.
Taxi Media	Company	Advertising	-	Defeat	Calls for increased promotion of healthy products instead.
Ubiquitous Ltd	Company	Advertising	Outsmart	Defeat	Urges the Mayor to jointly explore alternative approaches with advertising agencies.

^1^Business association = BA.

^2^Defined as: food/beverage production = companies who primarily manufacture foods or beverages for retail; Foodservice = (fast food) restaurant companies; food delivery = companies who primarily coordinate or execute the delivery of foods/beverages to customers; advertising = companies that primarily market/advertise products; cross-sectoral = spans various sectors.

^3^Membership of BAs, which responded the London Food Strategy consultation, identified via business associations’ websites.

ASA, Advertising Standards Authority; BA, business association; BSDA, British Soft Drinks Association; BTC, British Takeaway Campaign; FDF, Food and Drink Federation; HFSS, high in fat, sugar, and/or salt; IPA, Institute of Practitioners in Advertising; KFC, Kentucky Fried Chicken; PDM, Policy Dystopia Model; TfL, Transport for London.

### Discursive strategies

**Fig 2:**
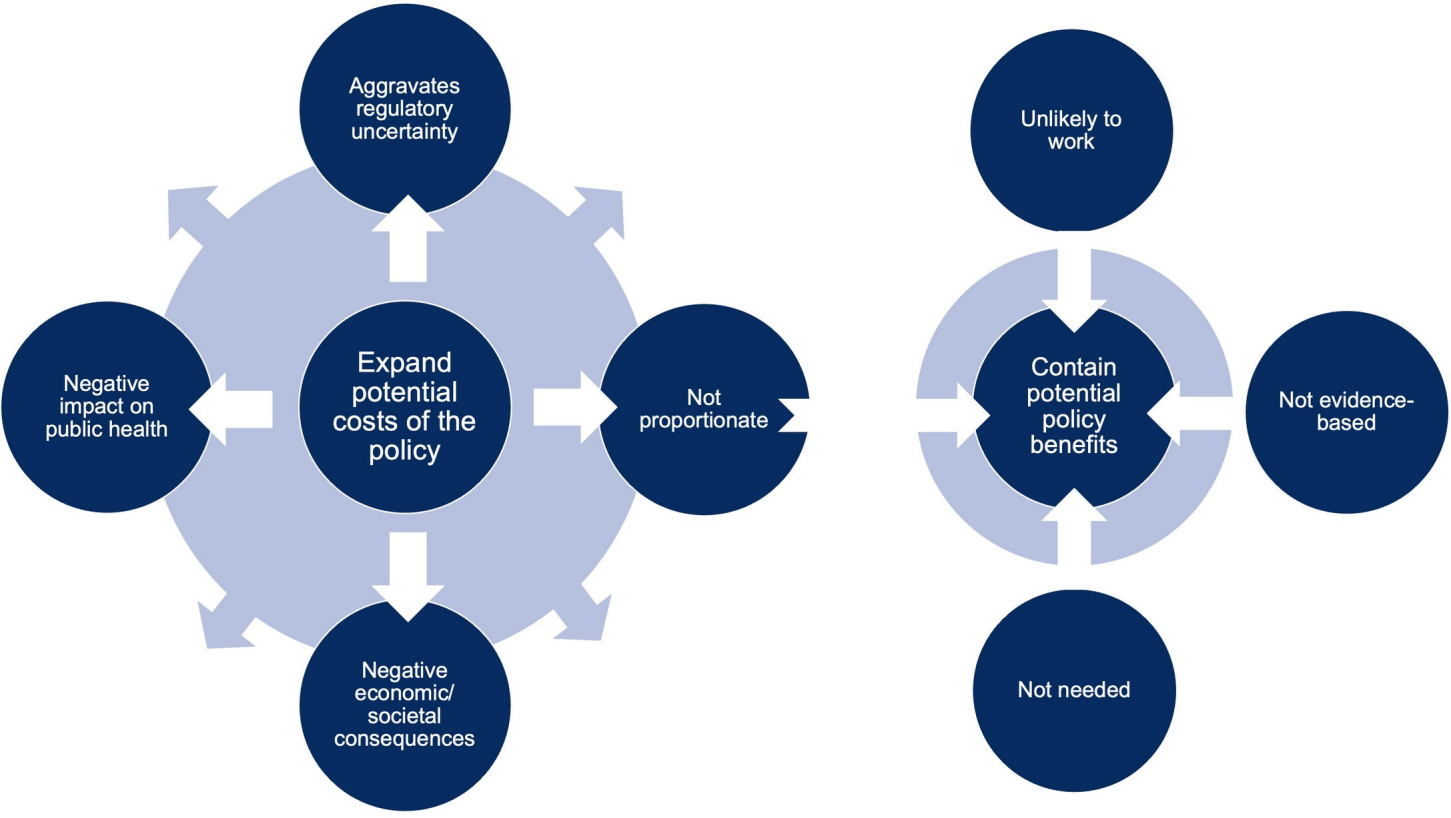
Discursive strategies used by food and advertising industry actors against the TfL advertising restrictions.

The arguments opposing the policy proposal were largely consistent across food and advertising industry respondents, although submissions varied in emphasis. The vast majority claimed support for the overall aims of the London Food Strategy but opposed the advertising restrictions. Fast food delivery company Uber Eats, for example, supported the Mayor’s plan on reducing obesity “in general” [103], and the fast food company McDonald’s claimed to understand the need for regulation “in essence” [104]; both companies then moved on to oppose the proposed advertising restrictions. To justify opposition to the policy, commercial actors sought to extend the possible costs, while simultaneously underplaying the potential benefits of the policy. Within these discursive strategies, they employed 7 key arguments (Fig 2). The language used in responses largely reflected a framing of obesity as a matter of individual choice, manifested, for example, in an emphasis on consumer choice and a rhetorical shifting of responsibility away from the companies that produce and market products. The discursive strategies we found were consistent with the PDM, bar one strategy, “unintended benefits to undeserving groups” (Table 1), which we did not identify.

#### Expanding or creating potential costs of the policy

*Unanticipated costs to economy and society*. Multiple respondents predicted that the advertising restrictions would have unintended negative consequences on the economy and wider society. Emphasising the economic importance of the food [103–109] and advertising [110–115] industries, many respondents warned of negative impacts of the proposed policy on TfL revenue [109,110,113,116–118], London’s economy [110,113,114,119], or wider society [111,113,114]. The fast food company Kentucky Fried Chicken (KFC), for example, cautioned that “[f]unds to support London’s transport network would be lost” [109], while advertising industry association Outsmart [113] cautioned of broader costs to the public good, claiming that the restrictions would

undermine our ability to invest in targeted measures to reduce childhood exposure to advertising and […] reduce our ability to commit current levels of funding to investments such as the installation of bus shelters and the provision of free WIFI.

They concluded that given “the severe impact a wholesale ban would have on our members, their customers and commercial freedom of speech as well as the knock-on adverse effects on the wider public interest, thorough consideration of alternative solutions […] is required” [113].

Advertising companies and associations highlighted potential costs to themselves [110,111,113–116,119,120] and their food industry clients [116,118], in particular smaller companies [116]. Outsmart [113], for instance, claimed that

[t]here would be a severe impact on our members’ business, as the affected outdoor advertising space could not simply be resold for alternative products. The likely reduction in revenue for the industry would be £375m over the next 5 years.

The fast food company Subway, as the only food industry respondent discussing cost to its own business, claimed that, as Londoners are harder to reach through TV and radio advertising, “the impact of the proposed changes to out-of-home [advertising][…] would be substantial, directly leading to a reduction in footfall and business performance” [121]. Respondents from fast food delivery companies also emphasised potential costs to smaller businesses [103].

Similarly, the policy was opposed on grounds it would be difficult to implement. Both fast food delivery company Just Eat and the business association British Takeaway Campaign warned that it would be challenging for smaller businesses to establish which of their products were HFSS, while advertising actors urged further consultation to improve the clarity of the policy [114,116,117,122]. Invoking parallel developments, the advertising restrictions were portrayed as adding to regulatory uncertainty in the context of Brexit [123], national obesity policy [110,113,122], a review by the Committee of Advertising Practice on their non-broadcast advertising rules [105,110,116,122], and the pending revision of the Nutrient Profiling Model [106,110,111,116,124], with some claiming the impact of the policy could not be assessed without the new model [105,107,113,122,125].

Several respondents argued that a ban on HFSS advertising should be rejected because it would be disproportionate [105,107,110,111,116–118,122,124,125]. Despite evidence on the scale of the problem clearly presented in the consultation document [126], the Food and Drink Federation, for instance, argued that it “would for the first time in the UK create a ban on food products being advertised regardless of the proportion of children and adults seeing the adverts, and would impose stricter regulations on food compared to alcohol” [107]. Similarly, and echoing concerns voiced by advertising businesses, the self-regulatory body ASA [120] argued that

most TFL properties at most times of day have an entirely or almost entirely adult audience. Banning HFSS ads on these properties, at these times wouldn’t do anything to reduce child exposure to HFSS ads and, therefore, an outright ban would appear to be completely disproportionate to the stated aim.

Though distinct in their use of the legal principle of proportionality, these arguments essentially connect a range of other claims discussed in this section. In summary, they convey the picture that restricting HFSS advertising would result in costs to the economy and society, which would not be justified by any potential benefits, as well as challenging the policy’s suitability—compared to less intrusive voluntary measures—and necessity. Proportionality arguments were also connected to denials of the evidence base underlying the policy (discussed below). The British Soft Drink Association, for instance, claimed that “academic research has consistently failed to establish a direct link between food and drink marketing and childhood obesity, therefore we are not convinced by the proportionality of further restrictions” [105].

*Unintended costs to public health*. Respondents claimed that the advertising restrictions might have an unintentional negative effect on public health by restricting the visibility of “healthier” alternatives [103–105,107,110,111,116,122–125,127,128], or even prompting a shift towards alcohol advertising [122], the latter contradicting Outsmart’s earlier claim that it would not be possible to resell advertising space. McDonald’s [104] warned that the policy risked “inadvertently increasing the obesogenic environment” because

only regulating marketing would have the unintended consequence of reducing the visibility of choice and restricting the information customers need to make the right choices for themselves and their families. […] restricting marketing in the wrong way will remove a key competitive lever and force business to consider changes to the price and quality of their food as the only remaining differentiators in the market.

Similarly, a number of respondents appealed for their own, “healthier” items such as fruit drinks [128], reformulated soft drinks [105], and dairy products [127] to be excluded from the advertising restrictions so as not to discourage their consumption or ongoing reformulation efforts, particularly in light of concerns that more of these products would be classed as HFSS under the draft updated 2018 Nutrient Profiling Model [129]. As of April 2021, the outcome of the 2018 consultation on the updated Model is pending.

#### Containing or denying potential benefits to public health

Commercial actors downplayed the potential benefits of the advertising restrictions, arguing they are unlikely to work, were not supported by evidence, and not needed. This undermining of potential policy benefits was rooted in arguments that childhood obesity is too complex to be appropriately addressed by advertising restrictions and instead required a “holistic approach” comprising a strong role for industry and nonstatutory interventions [109,113,122,123,125]. Ironically, the limited nature of the policy proposal, focused on out-of-home advertising, was used as an argument against regulatory action rather than in favour of more comprehensive measures. For example, some advertising industry respondents—notably all in the outdoor advertising business—warned that the policy would merely shift advertising into other, less regulated spaces, such as online [112–115,117,130].

Central to challenges to the policy’s effectiveness were discussions of evidence, invoked both as a rhetorical concept and by referring to specific sources. Despite compelling evidence that advertising influences children’s diets [15,131–133], food and advertising industry actors commonly claimed that empirical evidence in support of the advertising restrictions was absent or insufficient [105,107,110,113,116,120,125]. A number of respondents explicitly questioned the established link between advertising and eating behaviour [105,110]: The ASA, for instance, argued that “evidence consistently shows that advertising has no more than a modest influence on children’s food preferences,” citing no evidence to support that specific claim or anywhere in their submission [120].

Similarly, respondents portrayed the proposed advertising restrictions as redundant despite persistently high obesity rates [2,56], arguing that existing regulation, co-regulation, or self-regulation was sufficient or that the problem could be addressed through nonstatutory measures. Several ultraprocessed food industry actors underscored that they have responsible marketing measures in place and claimed that they do not advertise to children [103–105,108,109,123,134]. KFC, for instance, stated that they “do not and never will target children in [their] advertising, no matter the product or media channel” [109]. The Committees of Advertising Practice’s CAP and BCAP Codes were frequently invoked as sufficient [105,107,110,111,114,116,120,122–125], with the Food and Drink Federation, for example, arguing that “[t]he UK has one of the strictest advertising regulatory regimes in the world” [107]. In addition to highlighting ongoing voluntary efforts, many respondents endorsed further nonstatutory measures as a more desirable alternative, such as expanding existing self-regulatory practices [103,108,109,111,113,114,117,118,123,134] or harnessing advertising to promote healthy behaviour [104,109,111–114,117,119,123,130]. McDonald’s, for instance, made an appeal “to discuss how we can use our marketing skill to help the Mayor achieve his objectives” [104]. Correspondingly, a number of advertising industry respondents proposed a food equivalent to Drinkaware and GambleAware notices on alcohol and gambling adverts [113,114,117,118], linking to the existing TreatWise initiative founded by snack producer Mondelez International [135,136]. In a similar vein, using technological innovation to spatially and temporally restrict HFSS advertising was a popular alternative [103,106,108,109,111,113,114,117,118,122,134]. Domino’s, for example, explained that “[t]his might mean changing the time of day at which our ads are shown to avoid them being seen by children, for example, stopping them in late afternoon when children are on their way home from school” [134].

Respondents also criticised the effectiveness and appropriateness of the technical model underlying the proposed policy: the British Soft Drink Association, for example, stated that the Nutrient Profiling Model defines “products as HFSS, not as ‘unhealthy’ and therefore we do not believe the NPM [Nutrient Profiling Model] is the appropriate mechanism for determining whether food and drink products are ‘unhealthy’” [105].

### Instrumental strategies

Our analysis of instrumental strategies was based on consultation responses and correspondence between commercial actors and policymakers. The majority of interactions we discuss took place via the GLA and the associated Child Obesity Taskforce.

#### Coalition management

Coalitions between companies were the key technique we identified under coalition management. Several business associations that represent food and/or advertising companies responded to the London Food Strategy consultation [105,107,110,113,127]; as stated above, the majority of companies that opposed the advertising restrictions were members of at least one responding association. Notably, these associations did not represent any of the companies that supported the policy (S2 Table). Advertising companies coalesced around Outsmart, with several referring to [110,111,115–118,130] or even using sections from the association’s consultation response in their own statement [117]. One business group, the British Takeaway Campaign, was less transparent than others about who it represents. With a stated aim to “[champion] all those involved in the supply, preparation and delivery of takeaway food” [106], the group listed Just Eat as a member, but its submission and public website failed to clarify that it was established by a public relations agency, Newington Communications, on behalf of Just Eat in 2017 [106,137–139].

#### Direct involvement and influence

Although the GLA and TfL did solicit companies’ views, ultraprocessed food industry actors in particular endeavoured to increase their direct access to policymakers in several ways. These included requesting, arranging, and attending meetings, and attempting to join the Taskforce, which appeared to be seen as an important point of access.

**Fig 3:**
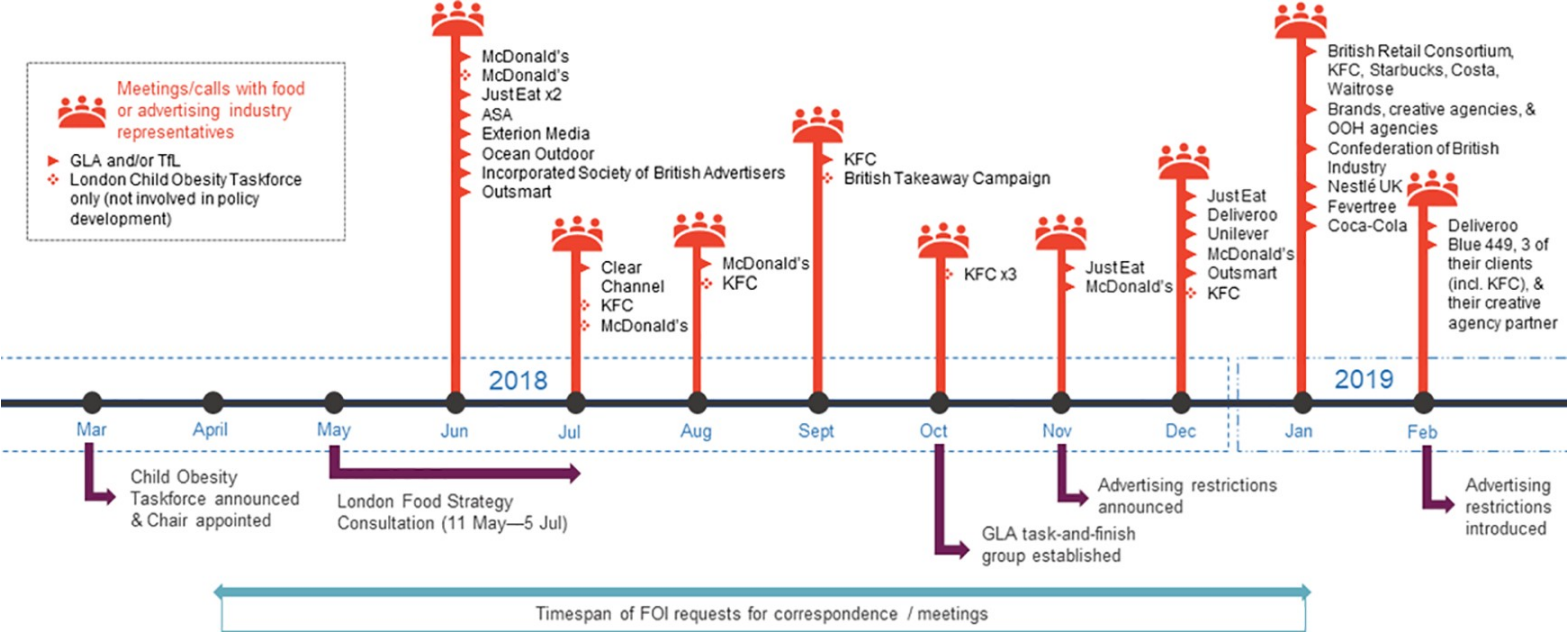
Timeline of the year leading up to the introduction of the TfL advertising restrictions and engagements with food and advertising industry actors during this time. Details can be found in S3 Table.

Food and advertising industry actors took part in the policy process by responding to the London Food Strategy consultation [64], which the GLA proactively encouraged some actors to participate in [140,141]. As shown in Fig 3 and S3 Table, GLA and TfL officials also held up to 8 meetings per month with industry stakeholders before the policy came into force. Some meetings were formally listed as part of the consultation process [64] while other, more informal interactions only emerged from correspondence between industry and officials: KFC, for instance, participated in at least 3 phone calls with members of the Taskforce and GLA staff and invited a Taskforce member to a “magical mystery tour” of London eateries and a tour of Brixton [141]. The same member also received an invite to KFC’s annual “Restaurant General Manager Fest” [141]. Notably, advertising industry body Outsmart expressed its disappointment at the level of involvement the GLA granted advertising industry stakeholders during the policy formulation process [142].

We also found evidence of attempts to establish access to policymakers, both longer-term and in the acute context of the advertising restrictions. McDonald’s, for instance, reached out directly to Taskforce members in April and to TfL in May 2018, asking “to meet for a coffee to have a chat about public health” even before the London Food Strategy consultation was officially launched. The company later applied to become a member of the Taskforce, although correspondence suggests that the application was delayed and thus not considered. Similarly, multiple emails suggest that KFC proposed “collaboration” with the Taskforce between August and October 2018, the nature of which we were unable to determine due to redactions. Public relations agencies also played a role in access-seeking: Just Eat was introduced to the Taskforce chair by Newington Communications, who also engaged with the GLA on behalf of the British Takeaway Campaign it helped establish [142].

#### Information management

Despite discursively contesting the evidence base supporting the policy, many commercial actors did not cite evidence in their consultation submissions. Where food and advertising industry actors shared or offered to share evidence with officials, they often had a role in its creation.

Sixteen of 27 submissions did not refer to any evidence in the context of advertising [103,104,109,115,118,119,121,123,124,128,130, 134] and those which cited evidence did so sparsely, only supporting selected points and largely relying on reports over peer-reviewed sources (see S4 Table for details). Across food and advertising industry submissions, the most frequently cited piece of evidence was a 2014 McKinsey Global Institute report without apparent food or advertising industry links [143].

In consultation responses and correspondence with officials, a number of industry actors shared research they had conducted or commissioned themselves [106,113,127]. For instance, emails suggest that KFC hired an agency to conduct research on “youth eating and snacking behaviour.” To our knowledge, this research remained unpublished. KFC invited GLA officials and Taskforce members to a research debrief at the offices of APCO Worldwide, a registered lobbyist for KFC [139], but the GLA responded to a Freedom of Information enquiry that they did not hold a copy of the presented research. Additionally, commercial actors sought involvement in evidence creation: McDonald’s, for instance, claiming that there was insufficient evidence for restricting advertising, offered support for “a London wide study of the causes of obesity” [104] through financing and expertise.

Respondents also shared information to favourably impact their image, such as corporate social responsibility initiatives in the areas of physical activity [104,105,123], food security [109,125], other social or environmental causes [104,111,113,114], and partnerships with public health bodies such as Public Health England [104,121]. For instance, Innocent raised its status as a “B corporation”—issued by a private organisation to corporations which “balance profit and purpose” based on ratings of social and environmental performance [144]—on several occasions [128,140] but did not explicitly mention being owned by soda multinational Coca-Cola [128] in its consultation response. Food companies frequently invoked “responsible advertising” initiatives, which challenged the need for the proposed advertising restrictions and offered further commitments as an alternative.

#### Legal strategies

Three advertising industry actors hinted at the potential for legal challenges, suggesting that this is something commercial actors may have been considering. Advertising industry body Outsmart disputed the 3-month implementation period, stating that “we believe it would need to be at least six months after any policy announcement is made to prevent the threat of legal action” [142]. In the consultation, Outsmart and 2 other advertising industry members cautioned that the proposed policy carried a higher potential for a legal challenge compared to self-regulatory approaches [113,117,118]. Talon Outdoor [118], for instance, stated that a “targeted,” self-regulated approach would “[reduce] the pressure for legal testing of the regulation”, while Kinetic Worldwide [117] claimed that

[s]elf-regulation is easier to manage from a regulatory perspective, as detailed and comprehensive regulations do not need to be drafted and enacted. Unilateral estatespecific [sic] regulation, as proposed here, would be under the same scrutiny as government regulation, but may have a higher potential for legal challenge.

## Discussion

In 2019, the GLA introduced a policy prohibiting HFSS advertising across the TfL estate, which caters to nearly 9 million Londoners and represents 40% of the city’s out-of-home advertising revenue [145]. To our knowledge, this is the first study to comprehensively examine industry efforts to oppose food advertising regulation in the UK. Overall, our findings broadly align with evidence of ultraprocessed food industry practices documented in other countries around the world [75–84] and draw comparisons with political practices of other unhealthy commodity industries [146]. As such, this study may have relevance for policymakers seeking to introduce restrictions to HFSS food and other unhealthy commodity advertising on publicly owned infrastructure, who want to prepare for the types of lobbying arguments and activities they may face.

### Key findings and links to the broader literature

We found that the majority of food and advertising industry actors opposed the advertising restrictions, with most advocating for voluntary measures or modifications to the policy in their own interest instead. The industry actors in favour of the policy were predominantly small businesses. Fast food companies such as McDonald’s and KFC, and the services that distribute their products—Uber Eats, Deliveroo, and Just Eat—all opposed it. In the sections below, we discuss the interlinking instrumental and discursive strategies we observed and contextualise them within the wider literature on corporate political activity.

Discursively, opposition was rooted in an underlying framing of obesity as a problem of individual responsibility. Through 4 key discursive strategies, commercial actors sought to simultaneously underplay the potential public health benefits of the policy and exaggerate its potential costs to the economy, public health, and society. These overarching strategies align with evidence on other unhealthy commodity industries [73,74,99]. One noteworthy difference in discourse between the food and advertising industries was that the latter commonly warned of costs to their own industry in consultation responses, whereas the former largely did not. In this respect, ultraprocessed food industry behaviour draws similarities with tobacco industry discourse, which tends to present policy costs as losses to the wider economy and society, rather to themselves [88]. One possible reason advertising actors used these arguments might be the lesser denormalisation and critical attention they have experienced as an industry, compared to large food corporations. This dynamic also reflects in arguments used by tobacco corporations in low- and middle-income countries, where their industry is less denormalised [147].

In parallel, food and advertising corporations and their representative associations used several instrumental strategies in attempts to gain access to the policy process, retain this access, and shape policy outcomes. Representatives of both industries were invited to participate in the consultation process by the GLA, and companies participated directly and indirectly through business associations. Attempts to increase this access included requesting formal and informal meetings with officials, for instance, applying for membership of or seeking collaboration with the Child Obesity Taskforce, even though the Taskforce was not involved in policy development or implementation. Opposing food and advertising industry actors engaged with the policy process directly and through business associations, while those who supported the policy were only represented directly. Evidence across unhealthy commodity industries suggests that such groups play a vital role in channelling the business voice in public health policy debates, allowing companies to be represented multiple times [88,148–151]. Coalitions like these thereby provide channels for amplifying corporate narratives and manufacturing a potentially misleading impression of substantial opposition [88].

We observed industry using inconsistent evidential standards across discursive strategies: Industry respondents challenged the substantial evidence underlying the advertising restrictions, yet only cited few sources, often reports rather than peer-reviewed evidence, to support their arguments against the advertising restrictions. As research from other public health areas shows, the instrumental and sometimes misleading use of evidence, both as an idea and via the citation of sources, is key to corporate attempts to legitimise arguments against regulation [152,153]. Commercial actors also shared (or offered to share) data and research they had sponsored with officials. This is potentially concerning considering a growing body of evidence indicating that industry-funded research is more likely to reach favourable or nonthreatening conclusions than independent research [154–158]. Similarly, “gifted” data may come with an implicit expectation of reciprocity [159].

Industry actors invoked corporate social responsibility initiatives and partnerships, potentially to convey a picture of the food and advertising industries as responsible societal actors. This aspect of information management may play a role in shaping the degree to which commercial actors are able to be involved in policymaking [160].

Lastly, some advertising industry representatives indicated that a legal challenge to the policy might follow. This also links to the discursive use of the legal principle of proportionality, which, at its core, requires that a measure must be necessary and must not exceed what is required to achieve its objective [161]. Specifically, industry actors claimed that the advertising restrictions were disproportionate on the basis that the burden on business and wider society resulting from the policy was not justified by its potential benefits to public health, and alternative (voluntary) measures would achieve the objective.

### Strengths and limitations

A key limitation of this work is that our sampling was restricted by the Freedom of Information processes we used to collect data. The Freedom of Information Act is subject to a number of exemptions, relating, for example, to public authority costs of retrieving data or commercial confidentiality [96], restricting the types and amount of data that can be requested and accessed. Investigations of this kind would be greatly facilitated by increased transparency of consultation processes, such as publishing consultation responses as standard practice, and an improved lobby register across all levels of public sector governance [162]. Because we focused on industry–policymaker interactions, we can only present a limited picture of the actual political activities that took place. This is particularly the case regarding the analysis of instrumental strategies, which benefits from using diverse types of data and having access to internal industry documents. For instance, our data are not suited to provide insights into techniques such as engagement with civil society or media: Advertising industry body Outsmart, for instance, has launched a “Get Smart, Outside” campaign focused on making the case against advertising restrictions [163]. We were only able to examine a time span of 10 months prior to the introduction of the policy due to limitations to how much information we could request. However, commercial actors may have sought to shape the policy at other stages, such as in its implementation. For example, we found evidence of advertising companies calling for an extension to the implementation period. Lastly, we were unable to establish if the activities discussed here had any actual impact on policy outcomes.

### Implications

Overall, the claims made by industry actors are not supported by independent public health evidence, which suggests that self-regulation of HFSS marketing is insufficient to prevent its established effects on children’s behaviour and food intake [15,20,133,164]. Respondents’ denial of the need for further advertising regulation was also based on claims that existing regulations are some of the strongest in the world, a notion challenged by civil society organisations that have consistently pointed out flaws in the existing UK system of self- and co-regulation [165,166]. Counter to warnings made by commercial actors, overall TfL revenue has slightly increased from 152.1 million in 2018 to 158.3 million since the advertising restrictions were introduced [58,167,168]. The public health impact of the policy is being explored through an independent evaluation that is underway as of April 2021. Although the restrictions alone are unlikely to have a measurable impact on obesity rates—as commercial actors pointed out—rejecting the policy on the basis that it only addresses one part of a complex problem reflects a “complexity fallacy” [169], which is common across unhealthy commodity industries and stands in the way of necessary incremental progress to overcome complex challenges.

The mere possibility of legal challenges, as implied by some advertising industry actors, can create regulatory or policy “chill,” leading to a public authority delaying, weakening, or abandoning a policy to avoid costly litigation [161,170,171]. Experiences from other unhealthy commodity industries indicate that the core aim of corporate legal threats and action is not to win a case, but rather stall or “chill” the policy process: Tobacco companies, for instance, publicly argued that health warning labels on cigarette packs contravened their trademark rights under international treaties, even though they had been given consistent legal advice that this was not the case [172]. There is a need for analysis of the legal barriers and opportunities with regard to local government regulation of food environments [173] to facilitate the disentangling of unsubstantiated legal claims and genuine legal hurdles. Similarly, proportionality claims, although not explicitly framed as legal arguments, do carry legal connotations. Thus, it is crucial that evidence underlying proposals for regulatory policies is framed in a way that demonstrates how the measure can contribute to public health, or obesity prevention more specifically, and no less restrictive, equally effective, alternative measure is available to achieve this objective [174,175]. Though arguably more proportionate, the voluntary and educational measures proposed by industry should not be seen as alternatives to regulation but implemented alongside advertising restrictions.

It is notable that the implemented policy includes a mechanism for advertisers to apply to TfL for products to be exempt from the restrictions. These guidelines were revised 4 months after their adoption, following initial media debate around the removal of a grocery advert containing butter, bacon, and jam, and reports of continued “junk-food” advertising across TfL [176,177]. A recent report by TfL states that 44 adverts were rejected on the basis of the HFSS advertising restrictions in 2019/2020, and of 81 valid exception applications, 27 were rejected [58]. The proximity of TfL to the companies affected by the advertising restrictions poses a potential threat to the organisation’s ability to be an independent arbiter in this matter: TfL’s partnerships in recent years have included companies that pushed back against the advertising restrictions, such as Lucozade Ribena Suntory [178] and JC Decaux [58]. Furthermore, the TfL staff members responsible for enforcing the application of the Healthier Food Advertising policy are from TfL’s advertising team and thus also responsible for meeting advertising revenue targets.

We highlight the strong but often overlooked role of the advertising industry and its coherence with the ultraprocessed food industry in opposing HFSS marketing restrictions. Further research is needed to critically examine the role they do and should play in the regulation of advertising. Similarly, delivery companies, which attempted to position themselves as technology rather than food companies, should not be overlooked as a potentially powerful player in public health policy. Notably, the ASA, which describes itself as “the UK’s independent advertising regulator” [179], was among the organisations opposing the TfL advertising restrictions, aligned in its position and arguments with the wider advertising industry.

## Conclusions

We uncovered substantial and comprehensive efforts by food and advertising companies to lobby against TfL’s advertising restrictions. As the UK government develops further restrictions on HFSS marketing to children, opposing voices have echoed the arguments identified in this study, claiming that the regulations will not work [180], are neither evidence based nor needed [181], and will have negative consequences [182]. Given the potential consequences of commercial influence on dietary public health regulation, it will be important for public bodies to consider the ways in which they engage with industry actors [183]. Where engagement is deemed necessary, it is important to scrutinise the evidence underlying industry claims. This may be facilitated by integrating conflict of interest disclosure and structured reporting of evidence into consultation processes.

## Supporting information

S1 Table Freedom of Information requests and responses. (DOCX)

S2 Table Excluded food/advertising industry and nonindustry actors and their positions on the advertising restrictions. (DOCX)

S3 Table List of meetings between officials and food/advertising industry representatives. (DOCX)

S4 Table Use of evidence in consultation submissions. (DOCX)

## Abbreviations

ASA: Advertising Standards Authority
GLA: Greater London Authority
HFSS: high in fat, sugar, and/or salt
KFC: Kentucky Fried Chicken
PDM: Policy Dystopia Model
TfL: Transport for London
