# Supplementary material for: Corporate political activity in the context of unhealthy food advertising restrictions across Transport for London: A qualitative case study
Source: PLoS Med. 2021 Sep 2;18(9):e1003695. doi: 10.1371/journal.pmed.1003695 (PMC8412307; doi:10.1371/journal.pmed.1003695)
Supplement: S1 Table — (DOCX) [file pmed.1003695.s001.docx]

**S1 Table: Freedom of Information requests and responses.**

| Public authority | Information requested | Request date—response date | Rationale | Outcome |
| --- | --- | --- | --- | --- |
| GLA | 69 priority London Food Strategy consultation responses, minus those which do not address the advertising ban | 14 May 2019—27 Jul 2019 (53 working days) | Initial request for all 149 responses was narrowed down on GLA’s suggestion to reduce time needed to contact the submitting organisations. A priority sample of 69 was identified, including food and advertising industry, and financially linked third party organisations (established through web searches). | Successful: 60 responses obtained with minor redactions (38 industry responses included in study). |
| GLA | 1.) Information on meetings with food/beverage/retail industry representatives regarding the advertising ban. 2.) Minutes and attendee lists for the following meetings: *Just Eat* meeting (20 Jun 2018), *McDonald's* meeting (4 Jun 2018).  3.) Correspondence between [named] officials and *McDonald’s, KFC, Just Eat*, *Deliveroo* (Apr 2018—Jan 2019). | 30 Jul 2019—11 Oct 2019 (53 working days) | The organisations included in the request for correspondence were highlighted in informal expert consultation as particularly active. Minutes for meetings with two of these actors (listed in the LFS consultation report) were requested, and a broad request was included to capture unlisted meetings. | Partially successful: No meeting minutes held, but dates and (anonymised) attendees of other meetings were released. Correspondence was obtained with substantial redactions.^1^ |
| PHE | Correspondence between [named] officials and *McDonald’s, KFC, Just Eat*, *Deliveroo* (Apr 2018—Jan 2019). | 13 Aug 2019—11 Sept 2019 (21 working days) | PHE officials were involved in policy development. The organisations included in the request for correspondence were highlighted in informal expert consultation as particularly active. | No information held. |
| TfL | **1.)** Minutes and attendee lists for the following meetings: *Just Eat* Meeting (20 Jun 2018), *McDonald’s* Meeting (4 Jun 2018). **2.)** Correspondence between TfL senior staff involved in the development of the advertising ban and *Coca Cola, Nestlé UK, Deliveroo, McDonalds, KFC, Just Eat* (Apr 2018—Jan 2019). | 15 Oct 2019—10 Jan 2020 (61 working days) | Meeting minutes for Just Eat/McDonald’s were requested TfL was the organiser, and GLA did not hold any minutes. | Partially successful: Correspondence obtained with minor redactions (none received with KFC). No meeting minutes held. |
| GLA | **1.)** Correspondence between [named] officials and *Coca-Cola, Innocent, Nestlé UK, Unilever, the Food & Drink Federation* (Apr 2018—Jan 2019). **2)** Minutes for the 1 January 2019 meeting with *Nestlé UK*. | 25 Oct 2019—22 Nov 2019 (21 working days) | Correspondence was requested for major food and beverage industry actors who had participated in the LFS consultation and meetings. Based on a previous request, the Nestlé meeting was the only one GLA indicated minutes were taken. | Successful: Correspondence obtained with minor redactions. No correspondence held for FDF. |
| GLA | Research on ‘youth eating and snacking behaviour' by *KFC* | 10 Mar 2020—14 Apr 2020 (24 working days) | GLA officials were invited to a briefing on this research by KFC (from previous requests). | No information held |
| GLA | Correspondence between [named] officials and the *Advertising Standards Authority, APCO Worldwide, Outsmart, Headland Consultancy, British Takeaway Campaign (BTC),* *PepsiCo* (Apr 2018—Jan 2019) | 2 Jul 2020—7 Aug 2020 (27 working days) | Expansion of correspondence data collection to key advertising actors (identified from consultation) and two PR agencies linked to KFC/Just Eat. BTC was included due to links to key player Just Eat. | Partially successful: Some correspondence received (no data for APCO, ASA, PepsiCo & Headland) |
| ^1^An internal review was requested to challenge these redactions based on section 43(1) & 43(2) and section 41 Freedom of Information exemptions. The redactions were upheld. | | | | |
