# Supplementary material for: Corporate political activity in the context of unhealthy food advertising restrictions across Transport for London: A qualitative case study
Source: PLoS Med. 2021 Sep 2;18(9):e1003695. doi: 10.1371/journal.pmed.1003695 (PMC8412307; doi:10.1371/journal.pmed.1003695)
Supplement: S2 Table — (DOCX) [file pmed.1003695.s002.docx]

**S2 Table: Excluded food/advertising industry and non-industry actors, and their positions on the advertising restrictions.** This list is based on the Freedom of Information responses we received, thus the non-industry respondents included here only represent a sample of other actors in the food space.

| Submitting organisation | Type | Sector^1^ | Position toward advertising restrictions | Statement on advertising restrictions^2^ |
| --- | --- | --- | --- | --- |
| Association of Convenience Stores | Business association | Food/ beverage retail | Not discussed | No discussion of advertising restrictions. |
| UK Hospitality | Business association | Foodservice | No clear position taken | “UKHospitality represents hospitality businesses, rather than advertisers or producers, but some of our membership advertise in a number of sites including TfL premises. We look forward to engaging with the upcoming consultation announced in this strategy, however we do have concerns around the definitions as to what is ‘healthy’ or ‘unhealthy’ in terms of proposed banned advertising (as this could capture a whole host of products unnecessarily), and we would like to see more clarity around this going forward.” |
| Beautiful Ornate Pieces Ltd | Company | Food/ beverage production | Adopt (or strengthen) | “If the only option is something unhealthy, fast and cheap then that's all they have as an option. If you are provided with an alternative then it makes your choice even more better” |
| CADA Design | Company | Advertising | Adopt (or strengthen) | “Advertising has a huge behavioural impact on us, and children are particularly susceptible. Advertisements are designed to nudge us toward spending money, and when public transport in London is free for children, they should not be forced to see unhealthy food choices.” |
| FoodExchange | Company | Other (venue) | Adopt (or strengthen) | “Personally I like the idea and think it would be a step in the right direction but being realistic there is an impact that has to be considered, “financially”” |
| Fruit Magpie | Company | Food/beverage production | Adopt (or strengthen) | “If it improves healthy food choices this can only do good” |
| Hep. instanbul vienna | Company | Other (consulting) | Adopt (or strengthen) | n/a |
| Jamie Oliver Limited | Company | Foodservice | Adopt (or strengthen) | “Yes! It’s a fantastic, world-leading and evidence-based move that signals the mayor is putting child health first. The world's biggest poster advertising estate on the planet will now be a force for good for the 30 million journeys a day that it serves. The evidence is clear: there is a link between food promotion and children’s food preferences, what they buy and what they eat. Advertising also influences how much children eat, and can lead to them ‘pestering’ parents to buy unhealthy products. Public Health England concluded in its review that “marketing is effective in influencing the purchase and consumption of high sugar foods. […] Transport for London has one of the world’s most valuable advertising estates. Just as TfL kick-started the fight against tobacco ads 30 years ago, the mayor has now done the same with junk food. It’s brilliant that this ban includes brand and directional advertising, too. Do we really need all London schoolchildren to know how far away they are from the Golden Arches at all times of the day? And for kids exiting tube stations to be immediately nudged into going to the McDonald’s down the road before they’ve even had a chance to make a choice about what they actually want to eat? This is a big stride forward for public health that generations.” |
| Karma - Good Food Shouldn't Be Wasted | Company | Food/ beverage other | Adopt (or strengthen) | n/a |
| London Farmers Markets | Company | Food/ beverage retail | Adopt (or strengthen) | “Parents need help to introduce children to healthier preference and eating habits. This builds on, and goes further than, existing precedents in other European cities, such as Amsterdam, where since 1 January 2018, no junk food ads likely to appeal to children or teenagers are allowed to appear anywhere on the municipal subway system.” |
| Maida Hill Place | Company | Food/ beverage other | Adopt (or strengthen) | “We work in an area of deprivation; Type 2 diabetes, obesity, coronary heart disease and poor dental health are rife. Poverty is at the heart of this - education around healthy eating is crucial. We think a ban on unhealthy food is a good idea but also think more needs to be done about healthy alternatives which are also affordable.” |
| Compassion in World Farming | NGO | Food/ beverage other | Adopt (or strengthen) | n/a |
| Global Aquaculture Alliance | NGO | Other (certification) | Adopt (or strengthen) | “Eating unhealthy food should not appear as mainstream” |
| Ecolocal on behalf of the Sutton Food Forum | Partnership | Food/ beverage other | Adopt (or strengthen) | “We strongly support the Mayor’s proposal to ban advertising of food that is not healthy across the TfL estate. This also gives a good message to other organisations and authorities.” |
| Good Food Greenwich | Partnership | Food/beverage other | Adopt (or strengthen) | “This is an excellent and ambitious idea and we fully support it. We would like to add that the ban should include adherence to the International Code on Marketing of Breastmilk Substitutes to protect and support breastfeeding and minimise advertising of breast milk substitutes that undermine breastfeeding. There should also be encouragement for Local Authorities to adopt the same approach through the advertising contracts they hold. Can we have proactive healthy advertising where the unhealthy ads leave space? Also improve the digital offer and develop London-level positive role models. (I don’t understand the latter point!) We think the ban should extend to alcohol too. The rationale for the advertising ban on junk food/drink seems to be to reduce children’s exposure to unhealthy messaging around food and drink, but surely adults are also susceptible to the same influences.” |
| Good Food Lewisham | Partnership | Food/beverage other | Adopt (or strengthen) | “We are very supportive of this; But, what will replace this advertising? We are concerned the gap will be filled with other ‘nuisance class’ advertising – can we advertise more healthy options to broaden the appeal of healthier food and info on Peas Please – could the veg cities campaign be included into TfL advertising? We are concerned the ban only applies to products, not companies, so the likes of Coca Cola and McDonald’s will still be able to advertise other products on TfL. We would like to see more on sponsorship. Will the ban extend to sponsorship of events? We think the Mayor should write guidance for Local Authorities of how to legislate procurement at events. We want the ban to include infant formula and baby foods. We would like to see an integrated Public Health campaign across London” |
| Alexandra Rose Charity | Registered charity | Food/ beverage other | Adopt (or strengthen) | “Our environment is saturated with messaging that is targeted at influencing the way that we consume. We need to do something bold to change the obesogenic environment and we think that this is an idea worth trying.” |
| Be enriched | Registered charity | Registered charity | Adopt (or strengthen) | “There is a strong correlation between the prevalence of food advertisements directed at children and their subsequent food preferences and choices. Research suggests that while unhealthy food advertisements don't necessarily directly result in childhood obesity, they may increase the likelihood of obesity as a result of poor food choices influenced by misleading and often manipulative advertising. Children and adolescents often turn to media and advertisements for guidance, and when the goal of the advertising is purely monetary rather than to promote the health and well-being of our youth, then a higher intervention is necessary.” |
| Borough Market | Registered charity | Food/ beverage retail | Adopt (or strengthen) | “We strongly welcome this policy, however note that it excludes advertising brand names where product is not displayed. We strongly recommend that the Mayor considers banning advertisements showing just a brand name, where that brand name is synonymous with food and drink that isn’t healthy. It is our position for example, that the McDonalds logo is so associated with Burgers and Fries that the imagery of the burger is ancillary.” |
| FareShare | Registered charity | Food/ beverage other | Adopt (or strengthen) | n/a |
| Flavour School | Registered charity | Food/ beverage other | Adopt (or strengthen) | “Junk food is so pervasive that chipping away at its all out assault on the senses, especially of children, is essential.” |
| Food Ethics Council | Registered Charity | Food/ beverage other | Adopt (or strengthen) | “Overall, we welcome the draft strategy document – particularly the breadth of issues covered (from household food insecurity to food growing, from Healthy start to public procurement and beyond) and the proposal of some bold interventions (e.g. banning junk food marketing in certain places), which – done well ‐ are much needed, given the scale of challenges we as society (and Londoners) face in relation to our food system.” |
| Food Foundation | Registered charity | Food/ beverage other | Adopt (or strengthen) | “The Food Foundation fully supports this proposal for the reasons outlined above. There will need to be careful work done on the scope of the ban. While the banning of adverts of specific foods which are high in fat, sugar and or salt is clear, it is not clear how the banning of brand only adverts will operate in practice. Specific thought will need to be given as to how e.g. McDonald’s salad would be handled or how a judgement would be made about whether a brand rather than a product were unhealthy (e.g. McDonald’s versus Tesco). Specific consideration will need to be given to directional advertising.” |
| FoodCycle | Registered charity | Food/ beverage other | Adopt (or strengthen) | “But this should also include alcohol. Alcohol is very unhealthy and destroys lives, especially those who may be in poverty, lonely or food insecure.” |
| Made in Hackney | Registered charity | Food/ beverage other | Adopt (or strengthen) | n/a |
| School Food Matters | Registered Charity | Food/ beverage other | Adopt (or strengthen) | “We whole-heartedly support the Mayor’s plans to consult on a ban on advertising of food and drink that is not healthy across the TfL estate. This will support children’s health and wellbeing by limiting their exposure to unhealthy messages.” |
| Covent Garden Market Authority | Statutory body | Food/ beverage retail | Adopt (or strengthen) | “The ban shows leadership by the GLA and sends a clear message about supporting good food in London.” |
| Hackney Food Partnership | Alliance | Food/ beverage other | Adopt (or strengthen) | “HFP strongly supports the proposed ban on junk food advertising on TfL property. It would be an important and ambitious step to tackle the power that the food industry has on influencing the high consumption of unhealthy food and drink. HFP believes that a high proportion of Hackney residents are likely to support the ban too, as demonstrated in the results of a Sugar Smart residents survey last October in which 69% supported action “to restrict the advertising and promotion of food and drink high in sugar”. Evidence shows that junk food advertising increases the consumption of unhealthy food and drink and that its influence starts at a very early age. Therefore the ban could help create a healthier food culture and encourage the food industry to both increase advertising of healthier food and reformulate unhealthy food, as demonstrated by the recent sugar levy. Such bold action also has the potential to set an example to other cities and national government in the UK and abroad to take action, particularly if the learning and impact is shared. […]” |
| Sustainable Food Cities Partnership Aberdeen | Alliance | Food/ beverage other | Adopt (or strengthen) | n/a |
| *^1^ Defined as follows: Food/beverage production = involved in the manufacture foods or beverages for retail; Food/beverage retail = involved in the sale of foods/beverages; Foodservice = fast food restaurant companies; Food delivery = companies who primarily coordinate or execute the delivery of foods/beverages to customers; advertising = companies which primarily market/advertise products; mixed/ other = does not fit into the other categories, or is a combination of organisations from various sectors.*  *^2^ n/a means that a respondent merely indicated ‘yes’ to the advertising restrictions.* | | | | |
