# Supplementary material for: Corporate political activity in the context of unhealthy food advertising restrictions across Transport for London: A qualitative case study
Source: PLoS Med. 2021 Sep 2;18(9):e1003695. doi: 10.1371/journal.pmed.1003695 (PMC8412307; doi:10.1371/journal.pmed.1003695)
Supplement: S3 Table — (DOCX) [file pmed.1003695.s003.docx]

**S3 Table: List of meetings between officials and food/advertising industry representatives.** We list public authority meetings with industry during the development of the TfL ad ban (before it came into force on 25 February 2019). Information on *formal meetings* is drawn from the consultation report and a GLA response to a request for information on *“meetings with food/beverage/retail industry representatives regarding the ban of unhealthy food advertising on the TfL estate”*. Information on all other meetings was completed from correspondence obtained through Freedom of Information.

| **Meeting date** | **Participating companies/ associations** | **Type** | **Participating public bodies** |
| --- | --- | --- | --- |
| 19 February 2019 | Deliveroo | Formal meeting, listed | GLA, TfL |
| 6 February 2019 | Blue 449 and 3 of their clients (including KFC) and their creative agency partner | Formal meeting, listed | GLA, TfL |
| 28 January 2019 | British Retail Consortium, KFC, Starbucks, Costa, Waitrose | Formal meeting, listed | GLA |
| 17 January 2019 | Brands, creative agencies and specialist OOH agencies (c120 representatives from various organisations) | Formal meeting, listed | GLA |
| 16 January 2019 | CBI | Formal meeting, listed | GLA, TfL |
| 15 January 2019 | Nestlé UK | Formal meeting, listed | Taskforce, GLA |
| 11 January 2019 | Fevertree | Formal meeting, listed | GLA |
| 3 January 2019 | Coca-Cola | Formal meeting, listed | GLA, TfL |
| 21 December 2018 | Deliveroo | Formal meeting, listed | GLA |
| 20 December 2018 | KFC | Brixton tour | Taskforce member & 1 other (redacted) |
| 18 December 2018 | Unilever | Formal meeting, listed | GLA, TfL |
| 17 December 2018 | McDonald’s | Formal meeting, listed | GLA, TfL |
| 11 December 2018 | Outsmart | Meeting/phone call | GLA |
| 10 December 2018 | Deliveroo | Formal meeting, listed | GLA |
| 5 December 2018 | Just Eat | Formal meeting, listed | GLA, TfL |
| 22 November 2018 | Just Eat | Phone call | GLA |
| 13 November 2018 | McDonalds | Formal meeting, listed | GLA |
| 31 October 2018 | KFC | “Magical mystery tour” of London eateries | Taskforce member & 1 other (redacted) |
| 19 October 2018 | KFC | Phone call | Taskforce member |
| 28 September 2018 | KFC | Meeting at City Hall | Taskforce member & others (redacted) |
| September 2018 | British Takeaway Campaign | Phone call | Taskforce member |
| 15 August 2018 | KFC | Phone call | Taskforce member |
| 2 August 2018 | McDonald’s | Phone call re Taskforce membership | GLA or Taskforce member (redacted) |
| 27 July 2018 | KFC | Phone call | Taskforce member |
| w/b 9 July | McDonald’s | Meeting re Taskforce membership | Taskforce member |
| 2 July 2018 | Clear Channel | Formal meeting, listed | TfL* |
| 26 June 2018 | McDonald’s | Meeting | Taskforce member |
| 20 June 2018 | Exterion Media | Formal meeting, listed | TfL* |
| 20 June 2018 | Just Eat | Formal meeting, listed | TfL* |
| 19 June 2018 | Advertising Standards Authority | Formal meeting, listed | TfL* |
| 11 June 2018 | Incorporated Society of British Advertisers | Formal meeting, listed | TfL* |
| 11 June 2018 | Just Eat | Formal meeting, listed | GLA |
| 6 June 2018 | Kinetic | Formal meeting, listed | TfL* |
| 5 June 2018 | Ocean Outdoor | Formal meeting, listed | TfL* |
| 4 June 2018 | McDonalds | Formal meeting, listed | TfL |
| 11 May 2018 | Outsmart | Formal meeting, listed | TfL* |
| **The indicated public body is named as the organiser of this meeting in the London Food Strategy consultation summary report. Other public authorities may have been present.* | | | |
