# Supplementary material for: Corporate political activity in the context of unhealthy food advertising restrictions across Transport for London: A qualitative case study
Source: PLoS Med. 2021 Sep 2;18(9):e1003695. doi: 10.1371/journal.pmed.1003695 (PMC8412307; doi:10.1371/journal.pmed.1003695)
Supplement: S4 Table — (DOCX) [file pmed.1003695.s004.docx]

**S4 Table: Use of evidence in consultation submissions.** For each submission opposing the TfL advertising ban, we identified whether any evidence was cited to support arguments in the context of the policy (as discussed under ‘discursive strategies’). Links to other policies or guidelines (including those of companies) which merely attest to the existence of a policy/guideline were not included as evidence.

| Submitting organisation | Evidence cited to oppose/challenge advertising restrictions? | Summary of evidence use in the context of the advertising restrictions |
| --- | --- | --- |
| Food and Drink Federation | Yes | Refers to [Buckingham (2009)](http://info.babymilkaction.org/files/DCSF%20report.pdf) to question link between advertising and obesity, [Dobbs/McKinsey (2014)](https://www.mckinsey.com/~/media/mckinsey/business%20functions/economic%20studies%20temp/our%20insights/how%20the%20world%20could%20better%20fight%20obesity/mgi_overcoming_obesity_full_report.ashx) to argue that media restrictions have a limited population impact, and [PHE (2015)](https://assets.publishing.service.gov.uk/government/uploads/system/uploads/attachment_data/file/470179/Sugar_reduction_The_evidence_for_action.pdf) / [PHE (2018)](https://assets.publishing.service.gov.uk/government/uploads/system/uploads/attachment_data/file/800675/Calories_Evidence_Document.pdf) to question the potential benefits of advertising restrictions. |
| Innocent | No | - |
| Just Eat | Yes | Cites [CEBR (2017)](https://cebr.com/reports/cebr-in-the-news/)—commissioned by Just Eat for the BTC, not available on BTC/CEBR websites at time of writing—to emphasise economic importance of the takeaway sector |
| Domino’s | No | - |
| KFC | No | Statistics are mentioned in the text, but the origin is unclear. |
| Lucozade Ribena Suntory | No | Only references other guidelines throughout the submission. |
| McDonalds | No | Statistics are mentioned but not referenced in the text. |
| PepsiCo UK | Yes | Cites [Dobbs/McKinsey (2014)](https://www.mckinsey.com/~/media/mckinsey/business%20functions/economic%20studies%20temp/our%20insights/how%20the%20world%20could%20better%20fight%20obesity/mgi_overcoming_obesity_full_report.ashx), [Livingstone (2004)](http://eprints.lse.ac.uk/21756/), and [PHE (2018)](https://assets.publishing.service.gov.uk/government/uploads/system/uploads/attachment_data/file/800675/Calories_Evidence_Document.pdf) to argue that advertising bans are unlikely to meaningfully affect obesity. |
| Subway | No | - |
| Uber Eats | No | - |
| British Soft Drinks Association | Yes | Cites data from [NHS Digital](https://digital.nhs.uk/data-and-information/publications/statistical/statistics-on-obesity-physical-activity-and-diet/statistics-on-obesity-physical-activity-and-diet-england-2018) and PHE [NDNS](https://www.gov.uk/government/collections/national-diet-and-nutrition-survey) to argue that juice is an important contributor to children’s fruit/vegetable intake, and the latter is too low – thus, these products should be exempt from advertising restrictions. Invokes statistics of unclear origin to emphasise the economic importance of the soft drinks sector. |
| British Takeaway Campaign (BTC) | Yes | Cites CEBR [Takeaway Economy report 2017](https://cebr.com/reports/cebr-in-the-news/) (commissioned by just Eat for the BTC, not available on BTC/CEBR websites at time of writing) and statistics from a Just Eat survey (not fully referenced). to emphasise economic importance of the takeaway sector. |
| Dairy UK | Yes | Refers to a variety of sources—PHE [NDNS](https://www.gov.uk/government/collections/national-diet-and-nutrition-survey), [PHE (2018)](https://assets.publishing.service.gov.uk/government/uploads/system/uploads/attachment_data/file/768368/NDEP_for_England_OH_Survey_5yr_2017_Report.pdf), [EFSA (2008)](https://www.efsa.europa.eu/en/efsajournal/pub/826), [FAO (2013)](http://www.fao.org/documents/card/en/c/5067e4f2-53f8-5c9a-b709-c5db17d55c20/), [RSC (2014)](https://doi.org/10.1039/9781849737562), [DoH (1991)](https://assets.publishing.service.gov.uk/government/uploads/system/uploads/attachment_data/file/743790/Dietary_Reference_Values_-_A_Guide__1991_.pdf)—to make the case for products containing >75% milk to be excluded from the advertising restrictions. |
| ISBA | Yes | Cites [DHSC (2018)](https://assets.publishing.service.gov.uk/government/uploads/system/uploads/attachment_data/file/718903/childhood-obesity-a-plan-for-action-chapter-2.pdf) to argue that current UK advertising regulations are already strong enough. |
| Advertising Association | Yes | Cites [Dobbs/McKinsey (2014)](https://www.mckinsey.com/~/media/mckinsey/business%20functions/economic%20studies%20temp/our%20insights/how%20the%20world%20could%20better%20fight%20obesity/mgi_overcoming_obesity_full_report.ashx), [Livingstone (2004)](http://eprints.lse.ac.uk/21756/), [Buckingham (2009)](https://webarchive.nationalarchives.gov.uk/20130321045318/https:/www.education.gov.uk/publications/standard/publicationDetail/Page1/DCSF-00669-2009), and [PHE (2018)](https://assets.publishing.service.gov.uk/government/uploads/system/uploads/attachment_data/file/800675/Calories_Evidence_Document.pdf) to argue that media restrictions have only limited impact on behaviour. Vague reference to “TGI” survey data to support the same claim. |
| ASA System | No | Refers to ‘the evidence’ to argue advertising only has a moderate influence on children’s food preferences but does not cite any sources in support. |
| Clear Channel UK Ltd | Yes | Cites [Dobbs/McKinsey (2014)](https://www.mckinsey.com/~/media/mckinsey/business%20functions/economic%20studies%20temp/our%20insights/how%20the%20world%20could%20better%20fight%20obesity/mgi_overcoming_obesity_full_report.ashx) to argue that measures such as education and portion control are “much more effective” than advertising restrictions. Mention of a report by Lord Darzi (without clear reference) in support of using out-of-home inventory to promote healthy behaviour. |
| Exterion Media UK Limited | Yes | Cites [DHSC (2018)](https://assets.publishing.service.gov.uk/government/uploads/system/uploads/attachment_data/file/718903/childhood-obesity-a-plan-for-action-chapter-2.pdf) to argue that UK advertising rules are among the strictest globally. [Dobbs/McKinsey (2014)](https://www.mckinsey.com/~/media/mckinsey/business%20functions/economic%20studies%20temp/our%20insights/how%20the%20world%20could%20better%20fight%20obesity/mgi_overcoming_obesity_full_report.ashx) cited to argue that education and information interventions are proven to be more effective than media restrictions. Statistics on children’s use of the London Underground are mentioned but not clearly referenced. Mention of a report by Lord Darzi (without clear reference) in support of using out-of-home inventory to promote healthy behaviour. |
| Institute of Practitioners in Advertising | No | Only references other guideline throughout the submission. |
| JC Decaux | No | - |
| Kinetic Worldwide | No | - |
| Outdoor Plus | No | - |
| Outsmart | Yes | Refers to Lord Darzi report ‘Better Health for London’ and [Dobbs/McKinsey (2014)](https://www.mckinsey.com/~/media/mckinsey/business%20functions/economic%20studies%20temp/our%20insights/how%20the%20world%20could%20better%20fight%20obesity/mgi_overcoming_obesity_full_report.ashx) to argue in favour of education campaign (instead of advertising restrictions). [Dobbs/McKinsey (2014)](https://www.mckinsey.com/~/media/mckinsey/business%20functions/economic%20studies%20temp/our%20insights/how%20the%20world%20could%20better%20fight%20obesity/mgi_overcoming_obesity_full_report.ashx) is also invoked to highlight media restrictions as one of the least effective obesity interventions. Refers to [Sheldon](https://www.bmj.com/content/361/bmj.k2534) (2018) and [OPRU](https://www.ucl.ac.uk/obesity-policy-research-unit/sites/obesity-policy-research-unit/files/what-learned-from-amsterdam-healthy-weight-programme-inform-policy-response-obesity-england.pdf) (2017) to state that the 12% reduction of childhood obesity in Amsterdam cannot be attributed to its Metro advertising ban. Also uses own estimates (without references) to argue that the advertising restrictions would have a negative impact on business. |
| Primesight Limited | No | Mention of a report by Lord Darzi (without clear reference) in support of using out-of-home inventory to promote healthy behaviour. |
| Talon Outdoor | No | - |
| Taxi Media | No | - |
| Ubiquitous Ltd | No | - |
